# Validation of the Korean Version of Nurses’ Moral Courage Scale

**DOI:** 10.3390/ijerph191811642

**Published:** 2022-09-15

**Authors:** Boram Lee, Younjae Oh, Eunhee Lee, Kyoung A Nam

**Affiliations:** 1 Graduate School of Health Science, Hallym University, Hallymdaehakgil 1, Chuncheon 24252, Gangwon-do, Korea; 2 Seoul North Municipal Hospital, 38, Yangwonyeok-ro, Jungnang-gu, Seoul 02062, Korea; 3 College of Nursing, Research Institute of Nursing Science, Hallym University, Hallymdaehakgil 1, Chuncheon 24252, Gangwon-do, Korea

**Keywords:** moral courage, professional ethics, nurses, validity, structural equation modelling

## Abstract

(1) Background: Research that examines moral courage has received a great deal of attention from scholars and practitioners in recent years due to the impact of moral distress experienced by nurses. Although it needs to identify the phenomenon related to moral courage among nurses, there has been a lack of instrumentation to investigate the quantitative aspects of moral courage among Korean nurses. This study aimed to test the validity of the Korean version of the Nurses’ Moral Courage Scale. (2) Methods: A cross-sectional study was conducted through convenience sampling of 243 nurses from two general hospitals in South Korea. (3) Results: The Korean version of the Nurses’ Moral Courage Scale was developed from construct validity evidence, including 12 items in four domains: ‘Compassion and true presence’, ‘Moral integrity’, ‘Moral responsibility’, and ‘Commitment to good care’. Concurrent validity was obtained according to the significant correlation coefficients among the variables: moral courage, moral sensitivity, and professional moral courage. (4) Discussion: Our research contributes to the knowledge and understanding of moral courage in the nursing context and encourages future researchers to conduct a quantitative analysis of moral courage among Korean nurses using the validated K-NMCS.

## 1. Introduction

Nurses, as healthcare professionals, have ethical duties to promote dignity and protect patients’ rights as advocates when faced with ethical issues. However, numerous nurses experience moral distress since they fail to act ethically according to their moral beliefs or ethical principles due to organisational situational constraints [1,2]. Thus, in recent literature, the promotion of organisations’ ethical attitudes has been emphasised to tackle ethical issues, and nurses are being encouraged to improve their moral courage at a personal level to apply their ethical values to actions [3,4].

Moral courage in nursing refers to ‘courage’ in exhibiting virtue ethics, which is one of the fundamental principles of nursing ethics [5]. Courage and moral courage have been interchangeably used in nursing ethics education in the last decades [6,7,8]. Lachman [9] explained that moral courage is ‘acting according to one’s ethical beliefs against fear’, which is essential for nurses to play roles as professional nurses. In other words, professional nurses are required to have moral courage following explicit ethical knowledge to promote human dignity, the central value of nursing, and to defend and practice the subject [10,11]. Based on Lachman’s definition of moral courage, nursing research has described philosophical considerations, and reviews the notion and practice of moral courage in nursing [9,12,13,14]. Several nursing scholars have also paid attention to moral courage, which is an essential element of ethical competence among nurses [4,15,16] Given the previous literature, an improvement in our understanding of the variables related to moral courage in nursing is required using a positivistic approach.

Recently, empirical research on moral courage has been on the rise in the field of nursing. The research studies mainly apply qualitative research methods to interpret the causes of the negative psychological responses to the ethical situations nurses or nursing students face [17], or to explore the deteriorating quality of nursing—both due to a lack of moral courage [4,18]. A few quantitative studies examined the relationship between moral courage and related concepts, such as moral sensitivity, moral distress, and the ethical climate among nursing students [19,20]. However, they employed the Professional Moral Courage Scale (PMCS) [21] developed for military officers, without validating it on a different population, such as nursing students. A validated instrument is needed to examine the general characteristics and tendencies of moral courage, and associations among concepts involving moral courage among nurses in South Korea.

Dr. Olivia Numminen and her colleagues [3] validated the instrument to measure moral courage among nurses and named it the Nurses’ Moral Courage Scale (NMCS). They redefined moral courage in nursing as ‘the nurse’s ability to rationally defend professional ethical principles and to act accordingly despite the anticipated or real adverse consequences of such action’. NMCS consists of four subdomains: ‘Compassion and true presence’, ‘Moral responsibility’, ‘Moral integrity’, and ‘Commitment to good care’. Based on their previous study, called the ‘concept analysis of moral courage in nursing’, the NMCS was developed by redefining the concept of moral courage using Aristotelian virtue, reflecting the epistemological attributes of moral courage in the nursing context. Although NMCS was validated theoretically and statistically in the context of Finnish nursing, moral courage derived from personal values can differ depending on sociocultural factors. That is, it is essential to test the applicability of NMCS internationally. Therefore, this study aimed to validate the Korean version of the NMCS (K-NMCS) based on the original version of the NMCS. Furthermore, we wanted to test its validity and reliability within the Korean nursing context.

## 2. Materials and Methods

### 2.1. Research Design

This study validated the linguistic and cultural adaptation of the K-NMCS using a strong program of construct validation [22]. This study validated the K-NMCS in three stages using Benson’s construct validation framework [22]. First, in the substantive stage, the translated and retranslated NMCS were validated. In this stage, the conceptual review on moral courage in nursing focused on conducting a turnaround, cultural and linguistic context to enhance the content validity of the translated and retranslated NMCS in Korean. Second, we tested the internal relations among observed variables in the structural stage. Here, the questions were linked to the structure of the construct by determining the degree of relation between the observed variables and the construct. Third, in the external stage, we tested relations among constructs by validating correlations of the K-NMCS with other variables.

### 2.2. Participants and Data Collection

We recruited the participants using convenience sampling—about 250 staff nurses were included from two general hospitals in the two major regions of South Korea. The general hospitals with at least 300 full-time registered nurses and diverse departments were selected, including general and critical care units. Upon consulting a professional statistician consultant, the required sample size was calculated at a significance level of α = 0.05, the power was set at 80%, the medium effect size = 0.15, and the number of relevant variables was determined using the G*power Program (3.1 version, IBM, Korea, Seoul, Korea). Adding a dropout and error range of 20% resulted in a necessary sample size of 250.

The 250 participants were assessed between 5 January and 12 March 2020. After filling out the questionnaires, the nurses were asked to seal the questionnaires in an envelope to maintain confidentiality. The researcher then collected the questionnaires during their visits to the hospitals. Only 244 questionnaires were returned, with a response rate of 97.6%, out of which an ambiguous and missing response was excluded. Hence, about 243 copies were deemed appropriate for analysis.

### 2.3. Instruments

#### 2.3.1. General Characteristics

The questionnaire used in this study was constructed based on the original instrument—the NMCS [3]. The scale consisted of items regarding the participant’s age, gender, educational level, position, work unit, and years of work experience in nursing. It assessed the self-evaluation of their knowledge levels of ethics and answered the sources of learning about ethics.

#### 2.3.2. Nurses’ Moral Courage Scale

The NMCS was developed by Finnish nursing scholars, Numminen and her colleagues [3]. We sought permission from Dr Numminen to validate the NMCS for the Korean nursing population. The NMCS is a 21-item scale in English, assessing the perception of ethical behaviour in four subdomains, (1) Compassion and true presence, (2) Moral responsibility, (3) Moral integrity, and (4) commitment to good care. The participants were asked to score each item on a five-point Likert scale, ranging from one (does not describe me at all) to five (describes me very well). A higher total score indicates higher self-assessed moral courage. The original research [3] reported the overall reliability as Cronbach’s alpha = 0.93. The reliability of the four subscales was as follows: Compassion and true presence = 0.81, Moral responsibility = 0.81, Moral integrity = 0.82, and Commitment to good care = 0.73.

#### 2.3.3. Korean Version of the Professional Moral Courage Scale

The Korean version of the Professional Moral Courage Scale (KPMCS) [23] was used to verify the convergence validity. The KPMCS [23] was validated for Korean nurses using the original version, PMCS, by Sekerka, Bagozzi and Charnigo [21]. Moon and Kim [22] permitted us to use the KPMCS. The KPMCS has a total of 12 items, and each item is scored on a seven-point Likert scale ranging from (never true) to seven (always true). The semantic scoring method is summative, and a higher score indicates a greater moral courage level. Moon and Kim [23] reported that the reliability of Cronbach’s alpha was 0.79, and our study found it to be 0.92.

#### 2.3.4. Korean Moral Sensitivity Questionnaire

We used the Korean version of the Moral Sensitivity Questionnaire (K-MSQ) [24], which was validated by Han, Ahn, Kim and Kim [24] from the original version, the MSQ by Lutzen, et al. [25]. Dr. Lützén and Dr. Han granted us permission to use the K-MSQ. The K-MSQ is a 27-item scale which includes two reverse-coded items. It comprises five subcategories of patient-centred nursing care, professional responsibility, conflicts, moral meaning, and beneficence. It uses a seven-point Likert scale ranging from one (strongly disagree) to five (strongly agree). The score ranges from 27 to 189, and the higher the score, the higher the moral sensitivity of the participants. Han, Ahn, Kim and Kim [24] found the scale’s reliability to be 0.89. The Cronbach’s alpha of the scale was 0.88 in our study.

#### 2.3.5. Data Analysis

- Substantive Stage: Translation and Cross-cultural adaptation of NMCS

We the translated NMCS using the back-translation method [26,27]. The NMCS was initially translated from English into Korean by three native Korean-speaking translators. The Korean version was then back-translated into English by an independent native English-speaking translator, blinded to the original version of the scale. All inconsistencies between the resulting English and the original versions were examined, and the Korean version was corrected accordingly. A Korean linguistic scholar then reviewed the corrected version.

Cross-cultural adaptation was evaluated with the content validity using the Index of content validity (item-level CVI; I-CVI) and the scale-level index CVI/universal agreement calculation method (S-CVI/UA) [26,28]. According to Polit and Beck [28], a scale with excellent content validity should be composed of I-CVIs of 1.00, with three to five experts and a minimum I-CVI of 0.78 for six to ten experts, and an S-CVI/UA of 0.80 or higher. Seven expert committees evaluated the content validity of the 21-item NMCS translated into Korean. Three rounds of evaluation took place until the final consensus was reached. In this study, the I-CVI and the S-CVI/UA showed an excellent content validity with 0.93 and 0.80, respectively. Finally, all the items of the questionnaire were reviewed by 11 staff nurses working at three general hospitals to confirm their readability.

- Structural Stage: Internal Relations among subdomains of K-NMCS

The data were analysed using the SPSS 25 program and the Amos 21 program. The psychometric evaluation of the K-NMCS included the Kaiser–Meyer–Olkin (KMO) measure of sampling adequacy and Bartlett’s test of sphericity to show whether the data were suitable for structure detection. The concept of moral courage can be influenced by cultural contexts and has recently been presented in clinical nursing research. To our best knowledge, research on the validation of the NMCS in other countries has been scant. Therefore, EFA was performed to check the factor structure of the measure and examine its internal reliability in the Korean context [29]. Confirmatory Factor Analysis (CFA) was performed to test the goodness-of-fit of the K-NMCS model and the convergent and discriminant validity.

According to Hair, et al. [30], convergent validity tests the degree to which a scale or set of measures accurately represents the concept of interest. A construct usually refers to a complex concept which includes several interrelated factors. The convergent validity was verified by computing the Average Variance Extracted (AVE) and Composite Reliability (CR) for every construct. To achieve the convergent validity, the value of the AVE should be 0.5 or higher and the value of CR should be greater than 0.7 [30,31].

Discriminant validity ensures that a construct measure is empirically unique and represents the phenomena of interest that other measures in a structural equation model do not capture [30]. To satisfy this requirement, each construct’s AVE must be greater than the Squared Multiple Correlation (SMC) with other constructs in the model [30,31], or the value of 1 should not be included in the 95% confidence interval of the correlation coefficient [32].

Internal consistency reliability was estimated using Cronbach’s alpha coefficients, and a minimum value of 0.7 is considered acceptable [33]. Corrected item-total correlation with the minimum criteria of r = 0.30 and inner-item correlation with an acceptable value of 0.3 ≤ r ≤ 0.7 were used in the item analysis of the K-NMCS.

- External stage: Relations among Constructs

The concurrent validity was examined to test correlations of the K-NMCS with other instruments, the K-PMCS and the K-MSQ. They were analysed using Pearson’s correlation. Concurrent validity is one approach to criterion validity that estimates individual performance on different tests approximately simultaneously [30].

#### 2.3.6. Ethical Considerations

The Institute Review Board of the university (HIRB–2019–089) approved this study before data collection commenced. The questionnaires were distributed to the nurses of each unit through the nursing departments of the two general hospitals that agreed to participate in the study. The participants were assured that they could withdraw from the study at any point without facing any repercussions and were guaranteed confidentiality and anonymity. Informed consent was obtained from the participants who voluntarily agreed to participate. If the participants could not deliver the questionnaire to the researcher at the time of its administration due to their working shifts, they were asked to seal it in an envelope to maintain confidentiality and hand it over later upon the researcher’s visit.

## 3. Results

### 3.1. Participants and Descriptive Statics

A total of 243 nurses from two general hospitals with diverse care settings and 19 wards completed the K-NMCS. Most participants were females (94.7%), in their twenties (60.5%), staff nurses (88.9%), and had associate’s/bachelor’s degrees (82.7%) in nursing. Most participants had over two years of work experience as full-time nurses (80.1%). Most participants evaluated themselves on their knowledge levels in ethics as satisfactory, good, or excellent (84.0%). This knowledge level was gained through their nursing practice (40.0%) and obtained from nursing training programmes, such as short-term courses, theme sessions, or continuing education (26.3%), rather than professional health care education programmes (23.1%).

### 3.2. Structural Stage: Internal Relations among Subdomains of K-NMCS

- Construct validity of K-NMCS

In the first step of the EFA of the NMCS, 12 items out of 21 were extracted with an eigenvalue of one or more and factor loading of 0.5 or more, based on statistical rationales; a minimum acceptable score for this test is 0.5 [34,35]. Regarding the exclusion of nine items, it was finally confirmed following expert panel discussions, which were conducted three times to consider the statistical findings and the difference between the Finnish and Korean nursing contexts. The distribution of items within each sub-scale of the 12-item K-NMCS was three items in the ‘Compassion and true presence’ subscale, two items in the ‘Moral responsibility’ subscale, four items in the ‘Moral integrity’ subscale, and three items in the ‘Commitment to good care’ subscale.

The 12 items analysed with the KMO test supported the adequacy of the sample for factor analysis (KMO = 0.876), and Bartlett’s test (χ^2^ = 1095.927, *p* < 0.001), which indicated the strong relationships among variables, confirmed the suitability for EFA. The four subscales explained a total of 68.64% of the 12 items and were the same as the item for each subscale of the NMCS. The item correlations (communalities) in the factor analysis for the K-NMCS ranged from 0.590 to 0.800 (Table 1).

**Figure 1:**
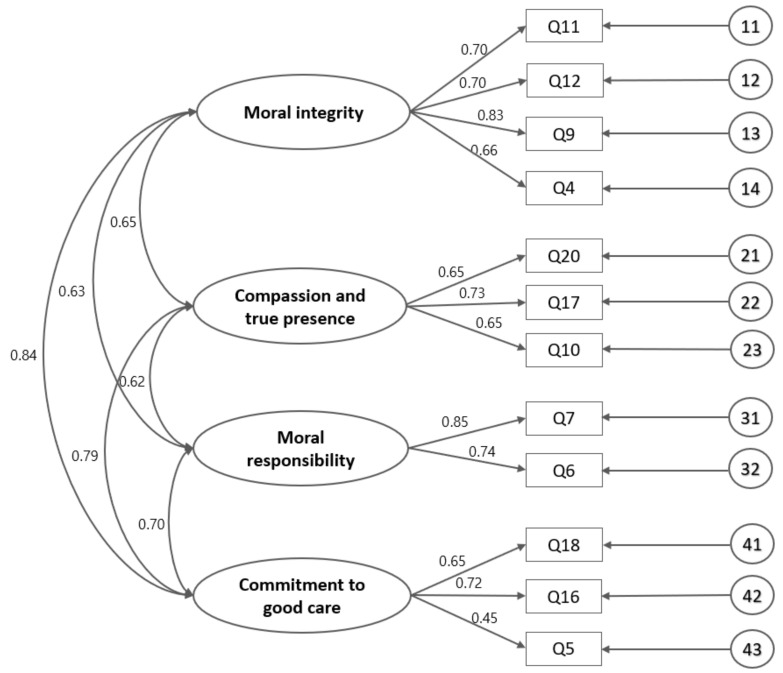
K-NMCS’s confirmatory factor analysis.

The CFA confirmed the construct of the K-NMCS (*n* = 243). The construct of the model was tested and is described in Figure 1. The fit of the model was determined by testing the hypothesised model using structural equation modelling (SEM) (Figure 1) and constructed with maximum likelihood estimations. Several criteria were used to examine the goodness-of-fit of the model, including nonsignificant chi-square statistics (χ^2^, *p*-value, degree of freedom), the CFI, the Tucker–Lewis Index (TLI), and the RMSEA. The chi-square test is an absolute test of model fit: if the probability value (*p*-value) is below 0.05, the model is rejected. For other indicators, the CFI takes into account the model fitting: it may range between 0 and 1, with values near 1 indicating very good fit [33,36]. The CFI value was 0.931, the TLI index was close to 1.0, and RMSEA values were <0.08, indicating a reasonable model–data fit [33,36]. All three criteria (CFI, TLI, and RMSEA) confirmed the goodness-of-fit of the model (Table 2).

- Reliability of K-NMCS

For the final 12-item K-NMCS, the total Cronbach’s alpha was 0.87, ranging from 0.62 to 0.81 for the four subscales, ‘Moral integrity’, ‘Compassion and true presence’, ‘Moral responsibility’, and ‘Commitment to good care’ (0.81, 0.72, 0.77, and 0.62, respectively), which was supported by the alpha of 0.60–0.70 indicating an acceptable level of reliability and 0.8 or greater indicating a very good level in social science research [37,38,39]. Item analysis revealed that all item-total correlations were higher than the minimum criteria (r ≥ 0.3). For all subscales, interitem correlations were at an acceptable level, based on the criteria of 0.3 ≤ r ≤ 0.7.

- Convergent and Discriminant Validity

The convergent and discriminant validities of the subscales were evaluated using CFA. The convergent validity was confirmed with the AVE being above 0.50 (range, 0.592–0.769) and the CR being above 0.80 (range, 0.813–0.904) [31] (Table 3). Discriminant validity was also confirmed; each subscale’s AVE was greater than the SMC with the other subscales, except in one case, in which the AVE (0.592) of F2 (Compassion and true presence) was lower than the SMC (0.6273) between F2 and F4 (Commitment to good care; Table 4). Additionally, the discriminant validity criterion was satisfied since the value of 1 was not included in the 95% confidence interval of the correlation coefficient (Table 2).

### 3.3. External Stage: Relations among Constructs

For concurrent validity, the relationship between the K-NMCS, K-PMCS [23], and K-MSQ [24] was verified using Pearson’s correlation as a statistical method. The 12-item K-NMCS and K-PMCS [23] showed a high correlation with 0.679 (*p* < 0.001), and the level of correlation between the 12-item K-NMCS and K-MSQ [24] (r = 0.348, *p* < 0.001) and between the K-PMCS [23] and the K-MSQ [24] (r = 0.449, *p* < *0*.001) were similar, establishing the concurrent validity [40] (Table 4).

## 4. Discussion

This methodological study was conducted to validate the K-NMCS, which examines moral courage in Korean nurses. As there were no theory-based constructs or validated items in Korea measuring the moral courage among nurses, we validated the K-NMCS based on the NMCS, developed by Numminen, Katajisto and Leino-Kilpi [3]. The K-NMCS also proved to be as valid and reliable as the original NMCS (Finnish version) and the Dutch version of the NMCS [3,15]. However, in the K-NMCS, nine items of the original scale were excluded due to the statistical criterion [34,35] and the differences in the nursing context between Finland and Korea, even though the four subscales notably explained a total of 68.64% of the 12 items and were the same as the items for each subdomain of the NMCS. The consistency of the items’ group between the K-NMCS and the NMCS can justify the validity of the original NMCS and support the generalizability of the instrument to measure the moral courage among nurses worldwide.

The nine items excluded from the NMCS were not significant in explaining the concept of moral courage among Korean nurses. Of these nine items, seven questioned the action to do the right things against situational constraints (e.g., the hospital power structure or ethical climate), and the others were about personal morality. These findings reveal that in the Korean context, doing the right things while confronting situational limitations might probably be perceived as foolhardiness instead of moral courage. Indeed, numerous Korean nurses felt too powerless to have moral courage against imperious and unethical hospitals or leaders’ attitudes, such as medical paternalism or collectivist and bureaucratic atmospheres [16,41]. Gallagher [42] pointed out that nurses’ moral courage can be highly influenced by situational constraints (e.g., the organisational climate or leadership), including personal moral courage. Thus, she argued that nurses’ moral courage should not be simply criticised and blamed without considering the situational constraints faced by them.

Organisations must support nurses’ ethical behaviours for them to act courageously as moral agents, practising ethical behaviours and professional ethics. It is evident from the previous studies [16,41,43,44] that several Korean nurses considered it was challenging to act as moral agents practising ethical behaviours. For instance, according to Kim, Oh and Kong [41], who explored ethical conflicts among Korean nurses, nurses were sceptical about executing ethically courageous behaviours against their organisations. Some nurses felt fearful and raged against unethical situations. However, their responses were affected by their learned sense of powerlessness and helplessness, due to repetitive experiences of seeing their courageous counterparts receive negative feedback from their colleagues and organisation [16,41]. Some nurses would prefer to leave their jobs than protest against their organisations because they could not play their roles as advocates [41]. As for professional autonomy, which is crucial in professional ethics, the level among Korean nurses was remarkably lower than in Western countries and in culturally similar East Asian contexts (e.g., Japan [45] and China [16]).

The ethical climate or leadership can be essential for nurses to practice ethical behaviour with moral courage. In dealing with ethical issues, person-oriented nursing values should be respected and supported by other professionals and their organisations. This interpretation can be supported by the authors of the original NMCS, Numminen, Konings, Claerhout, Gastmans, Katajisto, Leino-Kilpi and Dierckx de Casterlé [15], who also suggested that the NMCS should be developed through further research to reflect contextual factors such as the ethical climate or leadership [15]. Thus, further research is needed to examine the impact of organisational and other variables on moral courage among nurses in diverse care or cultural settings for the NMCS to reflect contextual factors.

### 4.1. Implications

This study highlights the evidence supporting the generalizability of the NMCS and enables other researchers to measure the moral courage level among Korean nurses, using the validated K-NMCS for comparative analyses with other culturally different countries or care settings. Further research based on the K-NMCS can foster a better understanding of moral courage in nursing and inspire the development of ethics programmes for nurses by providing fundamental data related to moral courage in the Korean nursing population.

### 4.2. Limitations

There are five limitations to this study. First, the generalizability of the results from the scale is limited as we used convenience sampling to validate the scale. Further studies using representative and diversified samples could improve the generalizability of the K-NMCS. Moreover, all the participants were females, as male nurses account for only 5.1% of registered nurses in South Korea [46]. Further research may be necessary to conduct studies with an adequate sample size of the male nurses, using stratified sampling to improve the generalizability of the scale. Second, we used EFA and CFA for the same groups, whereas those analyses have been traditionally recommended for different groups. However, according to other statistical perspectives, the same data set for EFA and CFA can be appropriate when the number of factors and the allocation of items to factors has not been well-known in evaluating psychometrics [7,47]. Third, differences in the number of items between the K-NMCS and NMCS may limit the ability to compare the data collected through scales internationally. However, the mean of moral courage and each subdomain can be compared with the means in the NMCS since the individual items in the four subdomains are the same as the NMCS. Fourth, the reliability of ‘Commitment to good care’ among the subdomains of the K-NMCS was acceptable; meanwhile, some empirical studies using the K-NMCS have shown its high reliability [48,49]. However, it is recommended that the reliability should be identified by other populations using the K-NMCS in the future. Lastly, our data were obtained using self-report questionnaires. Some participants might overreport their moral courage level as they might perceive it as one of the normative standards in nursing, hence, making them feel more ethical and professional. However, such concern can be lessened when psychometrically sound measures are employed [50].

## 5. Conclusions

The K-NMCS of four components is a reliable and valid instrument to measure nurses’ self-evaluated moral courage in diverse care settings in South Korea. Further validation studies in other cultural contexts and various care settings would shed light on understanding how nurses can ethically practice, motivated by moral courage as the norm to protect, maintain, and promote human dignity.

## Figures and Tables

**Table 1:** Pattern matrix of factor loadings of the K-NMCS items from the factor analysis (*n* = 243).

K-NMCS Sub-Scale and Items	Factors				
	Factor Loading				Communalities
	1	2	3	4	
**III. Moral integrity**					
If someone else* acts professionally dishonestly (e.g., steals medication from the ward), I bring it up for discussion	0.780				0.675
If someone else* tries to cover up an evident care mistake he/she has made, I bring it up for discussion	0.748				0.667
I bring up for discussion an ethical problem situation that arises in nursing care even if someone else* wants to remain silent about it	0.693				0.693
If someone else* acts unethically, I bring it up for discussion, even if I were to get negative feedback for it in my work community	0.684				0.590
**I. Compassion and true presence**					
Regardless of the care situation, I seek to create a genuine human encounter with the patient, even though a more superficial relationship would be easier for me		0.837			0.725
I support a suffering patient by being truly present for him/her, even if it were to lead me to encounter my own inner fears		0.740			0.676
Regardless of the care situation, I try to encounter each patient as a dignified human being, even if someone else were to disagree with my doing so		0.535			0.669
**II. Moral responsibility**					
I participate in the care team’s ethical decision-making regardless of someone else* disagreeing with the answer that I consider right			0.828		0.800
I participate in the care team’s ethical decision making despite the fact that ethical problem situations often involve uncertainty as to the right answer			0.810		0.785
**IV. Commitment to good care**					
I do not compromise on my patient’s right to good care even though someone else* were to bully me into doing so				0.635	0.658
I bring up for discussion the patient’s right to good care if someone else* compromises on adherence to the ethical principles of health care (human dignity, autonomy, justice, and justified care)				0.599	0.684
If I observe evident shortcomings in someone else’s professional competence, I bring it up for discussion				0.581	0.616

**Table 2:** Goodness-of-fit indices for the hypothesised model.

χ^2^	df	*p*	CFI	TLI	RMSEA	
					RMSEA	95% CI
120.589	48	<0.001	0.931	0.905	0.079	0.062–0.097

χ^2^, *p*-value: nonsignificant chi-square statistics; df: degree of freedom; CFI: comparative fit index; TLI: Tucker–Lewis index; RMSEA: root mean square error of approximation.

**Table 3:** Squared multiple correlation (SMC), average variance extracted (AVE), and composite reliability (CR) of the K-NMCS.

Subscale	SMC				AVE	CR
	F1	F2	F3	F4		
Moral integrity (F1)	1				0.702	0.904
Compassion and true presence (F2)	0.4212	1			0.592	0.813
Moral responsibility (F3)	0.3956	0.3881	1		0.769	0.869
Commitment to good care (F4)	0.7006	0.6273	0.4844	1	0.715	0.879

**Table 4:** Correlations among the K-NMCS, K-PMCS, and K-MSQ.

	Mean	SD	K-NMCS	K-PMCS	K-MSQ	Cronbach’s α
K-NMCS	3.26	0.52	1			0.872
K-PMCS	4.75	0.73	0.679 ***	1		0.916
K-MSQ	4.74	0.60	0.348 ***	0.449 ***	1	0.855

*** *p* < 0.001.

## Data Availability

Data will be available upon request from the corresponding author of the study.
